# Characteristics of Climate Change and Extreme Weather from 1951 to 2011 in China

**DOI:** 10.3390/ijerph15112540

**Published:** 2018-11-13

**Authors:** Chunli Zhao, Jianguo Chen, Peng Du, Hongyong Yuan

**Affiliations:** 1 Department of Engineering Physics, Tsinghua University, Beijing 100084, China; zhaochunli@mail.tsinghua.edu.cn (C.Z.); hy-yuan@tsinghua.edu.cn (H.Y.); 2 Institute of Public Safety Research, Tsinghua University, Beijing 100084, China; pdu@tsinghua.org.cn

**Keywords:** extreme weather, temperature, precipitation

## Abstract

It has been demonstrated that climate change is an established fact. A good comprehension of climate and extreme weather variation characteristics on a temporal and a spatial scale is important for adaptation and response. In this work, the characteristics of temperature, precipitation, and extreme weather distribution and variation is summarized for a period of 60 years and the seasonal fluctuation of temperature and precipitation is also analyzed. The results illustrate the reduction in daily and annual temperature divergence on both temporal and spatial scales. However, the gaps remain relatively significant. Furthermore, the disparity in daily and annual precipitation are found to be increasing on both temporal and spatial scales. The findings indicate that climate change, to a certain extent, narrowed the temperature gap while widening the precipitation gap on temporal and spatial scales in China.

## 1. Introduction

The global average temperature has climbed 0.85 °C since the industrial revolution, which resulted in remarkable warming in certain areas and impacts on flora and fauna, the ecosystem, health, public safety, and so on [1].

A number of research studies hold that climate change will cause some health problems [2,3,4]. For instance, Phalkey argued that there is significant correlation between climate change and child malnutrition [2]. Another study showed that 1 °C warming in the summertime could induce 1% demographic death, which reveals the potential threats of a sudden temperature change to the health of human beings [3]. In recent years, climate change also plays important roles in the deterioration of air quality in developing countries and regions [5].

Regarding the ecosystem and agriculture, Liu found that a 1 °C temperature rise could reduce wheat output by 5.7% (95% confidence interval ranges from 4.0% to 6.9%) [6]. A similar research study of Asseng showed 1 °C warming could lead to 6% reduction of wheat output, which is equivalent to one-quarter of wheat trade volume worldwide if no adaptive measures are taken [7].

Climate change, given a particular time and place, could trigger extreme weather or damaging events. For instance, floods, cryogenic disasters, and droughts are potential damaging weather events that could occur [8]. Due to global warming being accelerated by human activities, extreme climate events such as heat waves and torrential rains are becoming increasingly frequent [1,8]. Research studies predicted that if the global average temperature grows 2 °C above its level before the industrial age, human-induced climate change could trigger more than 40% torrential rain and 96% extreme high-temperatures [1].

Climate change and drastic fluctuations may lead to climatic disasters. For example, the freezing disasters may disrupt agriculture and transportation [9,10]. Better awareness of climate change on urban and regional scale are essential for actively responding to the adverse effects of climate change that is necessary for reducing fatalities and injuries to a large extent. Our research collects and arranges 60 years’ daily meteorological data in China and calculates the spatial distribution and temporal variation characteristic of climatic elements on a city scale. Based on the assessment, we formulate and describe the characteristics of climatic change in China. This paper presents preliminary work for clearing and delimiting the critical regions and cities for combating climate change and providing some data supports.

## 2. Materials and Methods

### 2.1. Materials

Based on 60 years of daily meteorological data in China, we extract the temporal and spatial variation characteristic of the temperature, precipitation, and extreme weather by using trend analysis methods and models. We summarize the spatial pattern and aggregation feature of climate change in China and probe the climate change effect and risk trait on a region and city scale.

**Figure 1:**
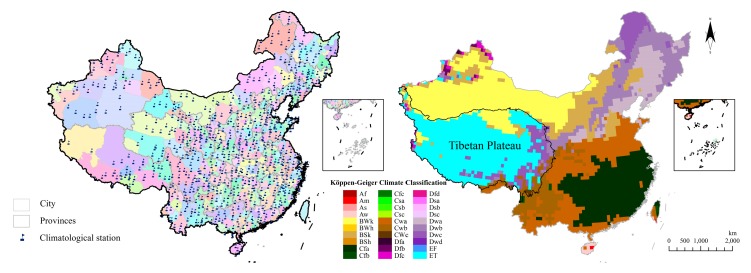
Selected meteorological stations and the climate Köppen of China. (The climate Köppen of China data is downloaded from the World Maps of Köppen-Geiger Climate Classification [12]).

The period of research is from the year 1951 to 2011. The meteorological elements include temperature, precipitation, extreme temperature, and extreme precipitation. The data is downloaded from the National Meteorological Information Center of China [11], which contains 756 meteorological station in China (Figure 1). In addition, Figure 1 demonstrates the climate Köppen of China. After basic cleansing, we use the ArcGIS10.1 (ESRI, Redlands, CA, USA) to interpolate the station data on the whole country scale and then analyze the climate element changing in time sequence by using the least square method. Annual and seasonal distinction is considered, which is divided into December to next February as winter, March to May as spring, June to August as summer, and September to November as autumn.

### 2.2. Method for Meteorological Data

After removing incoherent data sites and error logging data out of the meteorological site and database, we adopt the method of moving the average values for data preprocessing. Then we calculate the variation tendency and scope for annual and seasonal temperature and precipitation change. 

(1) Moving average. The research preprocesses the original meteorological data by moving the average values to bring down random fluctuation and uses the smoothed values to reveal and calculate the variation tendency. Every five years is a length for the moving average [13,14].

(1) $$\;f_{k}=\frac{1}{5}\;\left(y_{k-2}+y_{k-1}+y_{k}+y_{k+1}+y_{k+2}\right)\;$$

(2) Analyzing the variation tendency. A linear regression model is used to detect climate changes in trend by using the ordinary least squares (OLS) method. In this method, the linear relationship between a dependent variable (*y*) and an independent variable (*x*) is calculated below [15].

*y* = α + β*x* + ε   *x* = 1, 2, …, *n* (year) (2)

in which α is the intercept, β is the slope, and ε is the error term. When β > 0 (or β < 0), it stands that the evaluative feature is increasing (or decreasing).

(3) Fluctuation analysis. We employ the method of *CV* coefficient (coefficient of variation) to express the climate element’s fluctuation.

(3) $$\;CV=\frac{S}{\bar{x}}\times 100\%\;$$

in which the *CV* coefficient is the ratio of the standard deviation (*S*) and the mean value of *x*, which is a lower *CV* coefficient, indicates a minor degree of fluctuation.

### 2.3. Method for Extracting Extreme Weather

Since there are different definitions of extreme weather, correspondingly, there are a quantity of approaches for extracting extreme weather information, which contained the methods of the fixed threshold [16], the standard deviation threshold method, and the de-trended fluctuation analysis (DFA) [17,18]. This research adopts the extensive used percentile threshold method.

The percentile threshold method defines the threshold of extreme weather though a relative percentile-based temperature or precipitation indices. WMO has used this method to confirm the extreme weather threshold value (such as 1%, 5% as its maximum or minimum value) on its published climatological data, which exceeds the threshold value. This is considered climatic extremes. Percentile-based temperature/precipitation indices are emphasizing the need to remove the small probability of unusual weather from the climate mean state to predict the real extreme weather. Percentile-based indices are facing a wide geographical range. This method underlines the peculiarity of variations from region to region when evaluating the extreme weather.

This research study employs the extreme weather indices defined by the Expert Team for Climate Change Detection Monitoring and Indices (ETCCDMI) [19,20] and calculates extreme weather indices by the RClimDex (1.0) (Climate Research Branch Environment Canada, Downsview, Ontario, Canada), which is developed and maintained by Xuebin Zhang and Feng Yang at the Climate Research Branch of Meteorological Service of Canada. The indicators we select are shown in Table 1.

## 3. Results

### 3.1. Distribution and Change Characteristics of Temperature and Precipitation

#### 3.1.1. Distribution and Change Characteristics of Temperature

**Figure 2:**
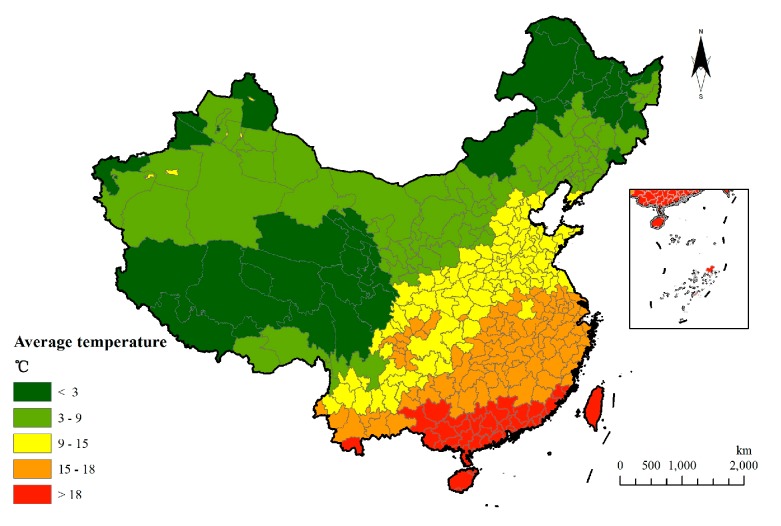
Distribution of average temperature in 60 years.

The spatial distribution of 60 years annual temperature showed that temperature progressively lowered from the southeast region to the northern region. There are large temperature differences between the north and south regions and the Tibetan Plateau has a low temperature center (Figure 2).

**Figure 3:**
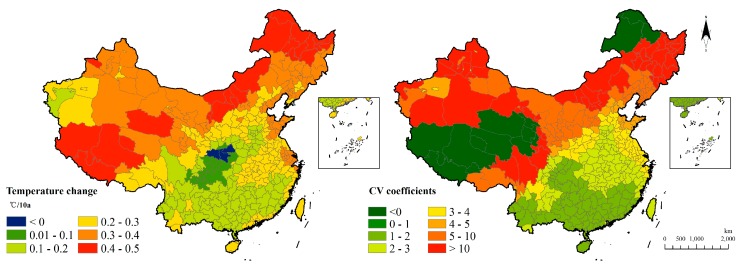
Temperature change and fluctuation in 60 years.

The temperature change during the 60-year period demonstrates a nationwide rising tendency except for a limited region in the middle of China (Figure 3). Specifically, the warmer southern region of China is confronted with a relatively small extent of the temperature rising while the colder northern region of China has a temperature that is increasing greatly. In other words, the results showed that the temperature rise tends to be more significant in regions with a lower average temperature (Figure 3). This phenomenon indicates that the northern region has more intense fluctuations than Southern China as a whole except for parts of the Tibetan Plateau and Northeast China.

**Figure 4:**
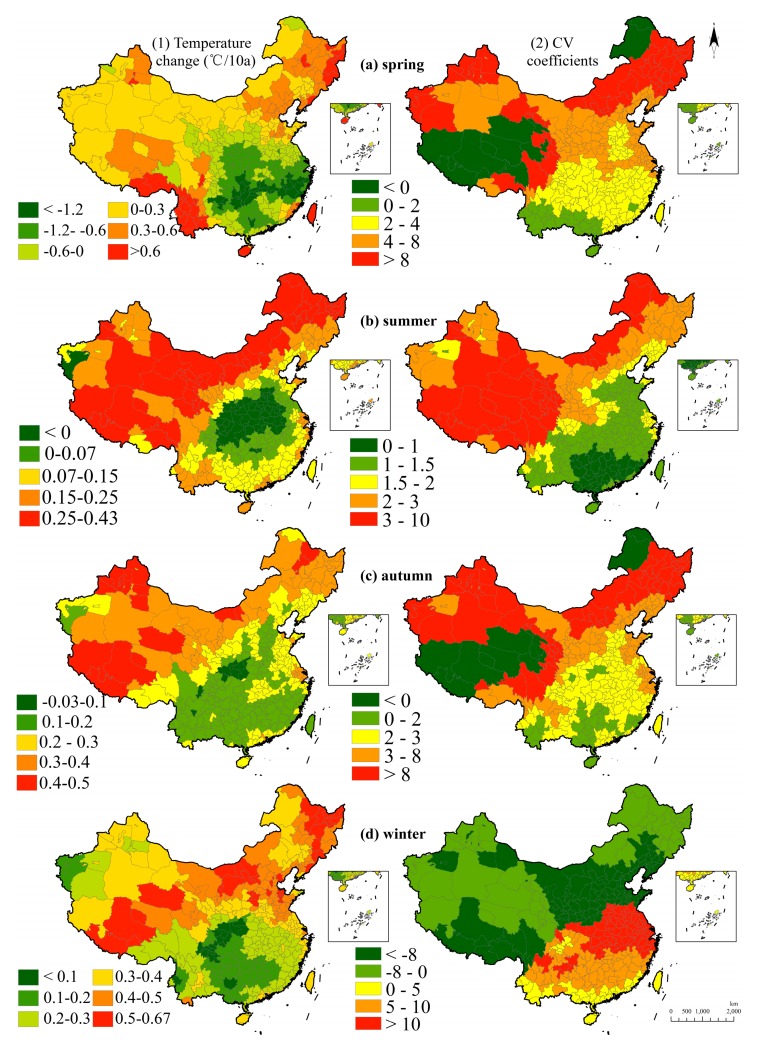
Seasonal temperature changes and fluctuations in 60 years. Note: (**a1**) is for the temperature change in the spring and (**a2**) is the CV coefficient. For the same period, (**b**–**d**) are corresponding to the temperature change and the CV coefficient in the summer, the autumn, and the winter.

Figure 4 illustrates a seasonal temperature change and fluctuation during the 60-year period. In the spring, the temperature decreased in Southern China and grew in the north and west regions, which indicates that cold regions are getting warmer and hot regions are cooling. In the summertime, the temperature demonstrates a rising tendency except in central China where a cooling process is observed. Furthermore, Hei Longjiang, Inner Mongolia, Ningxia, Gansu, Xinjiang, and Tibet are found to have a relative high rate of a temperature rise at 0.26–0.43 °C/10a. In the autumn, there is almost a uniform warming tendency except in several cities in the central region and a higher warming rate is observed in the northern region. In the winter, the temperature change is similar to that in autumn and the overall warming rate is higher than in the summer and in autumn.

#### 3.1.2. Distribution and Change Characteristics of Precipitation

**Figure 5:**
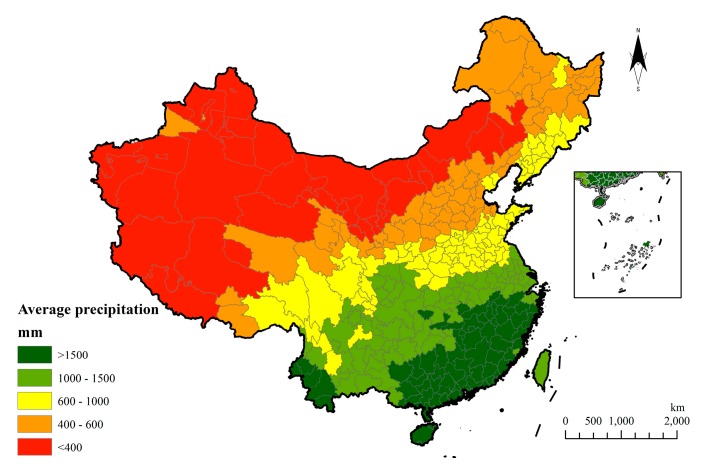
Distribution of average annual precipitation in 60 years.

The mean annual precipitation from 1951 to 2011 is shown below (Figure 5) and the spatial distribution characteristics are manifested as gradually increasing from the northwest to the southeast.

**Figure 6:**
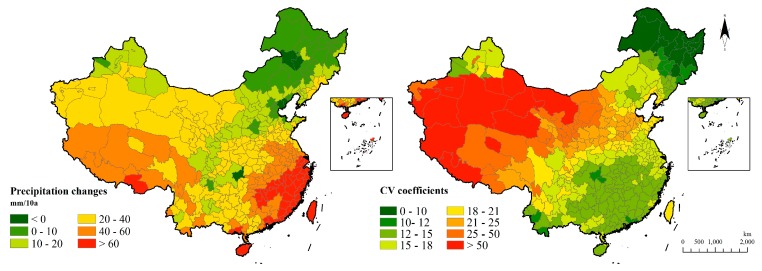
Precipitation change and fluctuation in 60 years.

Precipitation was found to have increased in most areas during the 60 years studied. It shows that the southeast region has a progressively increased tendency and it has the fastest increase, which is followed by the Tibetan Plateau with a gradient in (4.0, 6.0). A precipitation decreasing region is mainly in Northeastern China. In the perspective of precipitation fluctuation, the narrowest margin of precipitation distributes in the southeast and northeast region and the most fiercely fluctuation appears in the northwest and the Tibetan Plateau (Figure 6).

**Figure 7:**
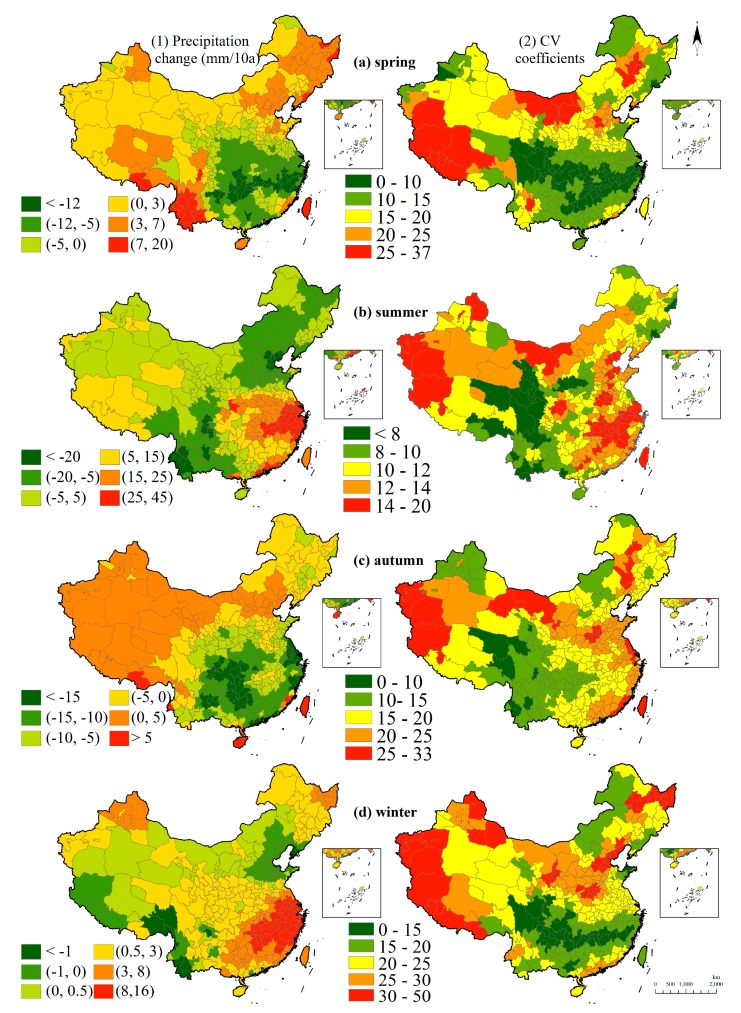
Seasonal precipitation changes and fluctuations in 60 years. Note: (**a1**) is for the precipitation change in the spring and (**a2**) is the CV coefficient. For the same outcome, (**b**–**d**) are corresponding to the precipitation change and the CV coefficient in the summer, autumn, and winter.

On a seasonal scale, the precipitation has a lessening tendency in the southeast region while increases are apparent in the southwest region in the spring. In the summer, only the southeast region is increasing in precipitation. The precipitation rate in the autumn rises in the northwest region and the Tibetan Plateau and decreases in the southeast region. Precipitation in the winter increases in most regions of China and the greatest growth appears in the southeast region (Figure 7). In the southeast region, results showed the precipitation levels are decreasing in the spring and autumn while increasing in the summer and winter, which may strengthen the centralization of annual precipitation. Increased exposure of rainfall and flooding in the summer or snow water in the winter may have negative impacts on production and living [21].

Seasonal fluctuation is found that the Tibetan Plateau and some northern parts of China are drastically fluctuating in terms of precipitation in the spring while the south region with abundant rainfall shows slight fluctuations. In the summer, the slight fluctuation region is focused on the middle-western region of China where there are insufficient water resources. In the autumn, rainfall fluctuation is drastic in Western China and slight in the middle-western region. Winter fluctuation shows drastic fluctuations in Northern China and slight fluctuations in the southern region.

### 3.2. Extreme Weather

#### 3.2.1. Extreme Temperature

We choose four indexes to express extreme temperature including the cold spell duration indicator (CSDI), the warm spell duration indicator (WSDI), the cool nights indicator (TN10p), and the warm day indicator (TX90p).

**Figure 8:**
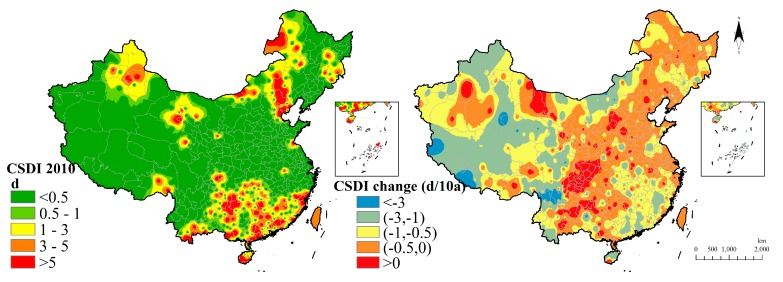
Cold spell duration indicator distribution and change.

CSDI shows that the southern region owned a long cold spell duration than the northern region in 2010. As for the 60 years’ variation, most region had a decreasing CSDI and parts of the middle region with the red color were increasing in CSDI (Figure 8).

**Figure 9:**
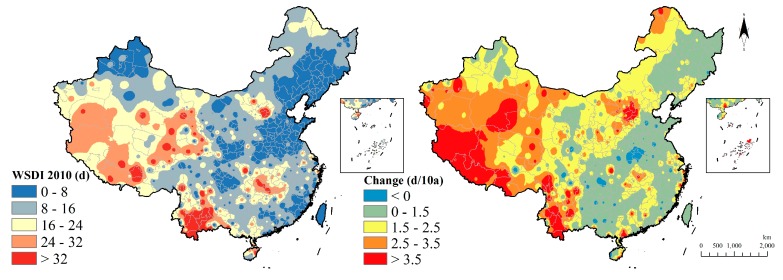
Warm spell duration indicator distribution and change.

WSDI in 2010 showed that the western region had a longer duration of warming than the eastern region and the high value region is in the southwest region (Yunnan province) exceeding 24 days. Additionally, 60 years’ variation manifested that almost the entire country showed an increased tendency in the WSDI except for a few regions with the western growth being faster than the eastern and the highest value being in south Xinjiang and north Tibet, which is similar to the research of Yao [22] (Figure 9).

**Figure 10:**
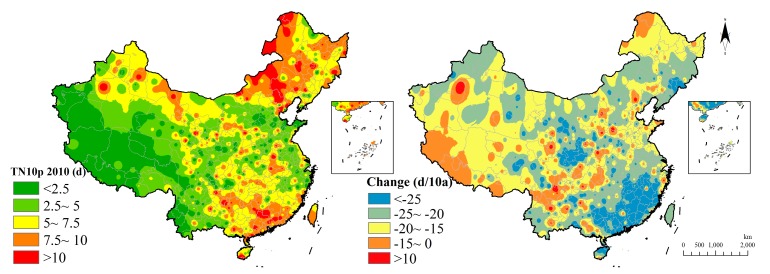
Cool nights indicator distribution and change.

TN10p shows a spatial gradient and retains longer in the eastern region than that in the western region in 2010. It keeps more than 10 cool nights in some southeast and northeast regions. Variation in 60 years told that the cool nights indicator in just few regions of the northwest are increasing while most regions are decreasing and the highest value occurs in the southeast region. It manifests that night temperature grew a lot during the past 60 years (Figure 10).

**Figure 11:**
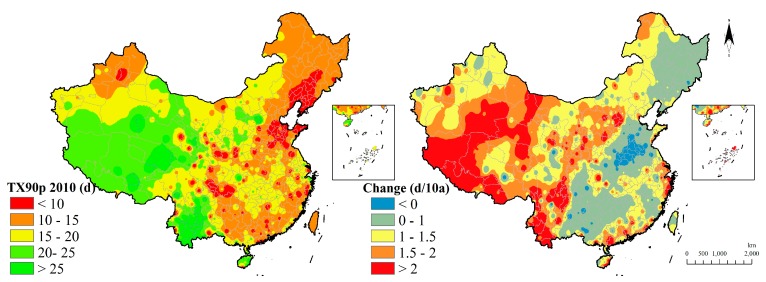
Warm day indicator distribution and change.

TX90p maintained that the western region has longer warm days than in the eastern region. Specifically, TX90p in the eastern region kept the values for 15–20 days while, in the western region, it is more than 20 days. In addition, the 60 years’ TX90p change showed that few regions in the middle-eastern region are decreasing and most regions have an increasing tendency. The western region increases more than in the other region (Figure 11).

#### 3.2.2. Extreme Precipitation

Extreme precipitation is described by four indexes of the consecutive dry days indicator (CDD), the consecutive wet days indicator (CWD), the heavy precipitation days indicator (R20), and the very wet days indicator (R95p).

**Figure 12:**
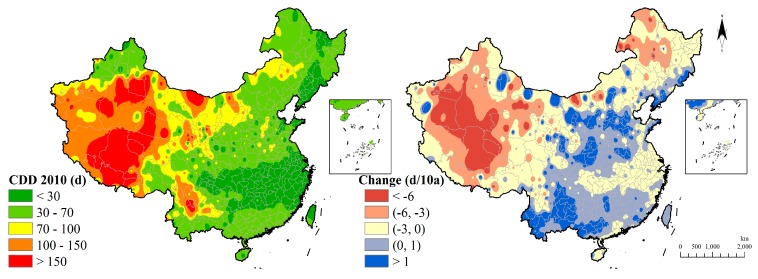
Consecutive dry days indicator distribution and change.

The distribution of CDD reveals that consecutive dry days are present in Xinjiang, the Tibet province, and the southwest region such as the Yunnan province. The CDD value showed more than 150 days of consecutive dry weather in 2010 in part of those regions. Change analyzing shows the CDD index is slowing down in the severe dry regions while aggravating in some of the middle and southern regions (Figure 12).

**Figure 13:**
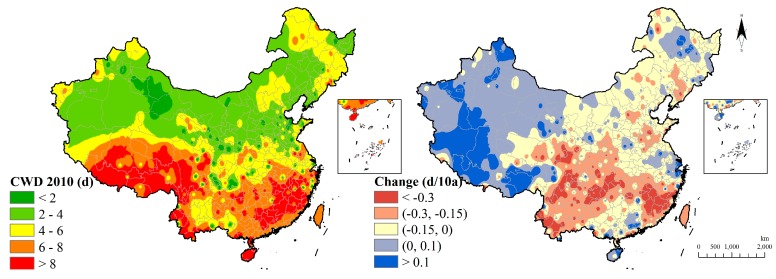
Consecutive wet days indicator distribution and change.

For the spatial distribution of CWD, South China and the Tibetan Plateau own the high value of CWD of more than eight days. Combined with the CDD, we can see that Tibetan Plateau is a temporally concentrated region of dry and rainy weather. The change of CWD performed a decline tendency in the southern region while having a growth trend in the Tibetan Plateau (Figure 13).

**Figure 14:**
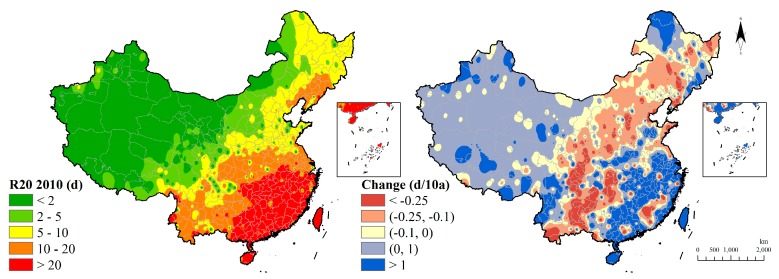
Heavy precipitation days indicator distribution and change.

The Southern China has a high value of R20, which kept more than 20 days in 2010 while the western arid region recorded less than three days, which shows a gradient sharp from the southeast to the northwest regions. The extreme precipitation in the southern region were strengthened in 60 years and the smaller area is the red region distributed from the northeast to the southwest regions (Figure 14).

**Figure 15:**
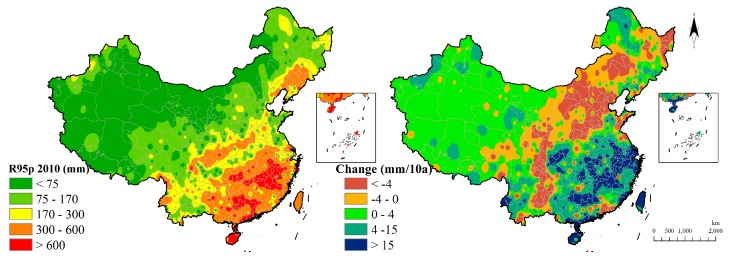
Very wet days indicator distribution and change.

R95p is an enhanced version of R20 and the southern region increases more dramatically in R95p (Figure 15).

## 4. Discussion

For temperature change and extreme temperature:

On the whole, a warming tendency is observed in all seasons nationwide (except for some central regions where temperature descended in the spring). Specifically, the northern region has a greater temperature rise than the south. The warming is faster in the winter and the spring than in the summer and autumn. Therefore, this phenomenon may lead to reduced gaps in temperature spatially between the north and south as well as temporally among the four seasons.

(1) Similar results are reported in some other works. Yan et al. [23] conducted a wavelet analysis for a day-by-day temperature change and fluctuation in Europe and China during the last 100 years and results showed that seasonal fluctuations lessened under the influence of global warming.
(2) In our research, the warm day indicator increased and the cool night indicator decreased, which indicates that extreme hot weather in the daytime is enhancing and extreme cold weather in the nighttime is weakening. Some research deemed that the latter indicator changes faster than the former, which leads to a narrower diurnal temperature variation [24,25,26,27].
(3) At the same time, a cold spell duration indicator is continuing to decrease in the western inland areas and increase in the eastern region while the warm spell duration indicator has the opposite trend. This phenomenon gives rise to a narrower regional temperature difference between eastern and western inland areas.

For precipitation change and extreme rainfall:

(1) On the whole, precipitation in the northern region is decreasing in the summer and autumn and, in the southern region, it is decreasing in the spring and autumn while increasing in the summer and winter. Precipitation changes faster in the southern region than that in the northern region. This change in the southern region are contributing to rainfall misdistribution between seasons. Annual variation is unevenly distributed in the southern region.
(2) The results show that extreme precipitation is strengthening in the southern region. This may contribute to reinforce the precipitation pattern of the “southern flood and northern drought.” Based on the GLM analyzing, Wang et al. considered that this phenomenon is mainly affected by the warming tendency on a large-scale [28].
(3) Combined CDD with CWD indicators, we can see that both of these two indexes are growing in the Tibetan Plateau despite the fact that this region is the high value center of these two indexes. This means that precipitation distributed concentration is on a temporal scale. In other words, the wet-season and dry seasons are more clearly divided in the Tibetan Plateau.

From above, it is observed that the gaps of the annual and daily temperature are reducing spatially from the south to the north and the east to the west even though these gaps remain relatively significant. In the meantime, the disparity between annual and daily rainfall in the north and south is growing. These findings indicate that climate change potentially narrowed the temperature gap and widened the precipitation gap on a temporal and spatial scale in China.

## 5. Conclusions

This research analyzes and discusses the temporal and spatial variation characteristic of temperature, precipitation, and extreme weather, which can draw a conclusion that, for a temperature change, a warming tendency is observed in all seasons nationwide and the spatial-temporal change shows temperature gaps spatially narrows between the north and the south as well as temporally among the four seasons. In addition, an extreme temperature shares the similar change trend, performance as a narrower regional temperature difference between Eastern and Western China, and a narrower diurnal temperature variation. For precipitation change, it shows as a misdistribution between seasons in the southern region. Extreme precipitation change emphasizes the precipitation pattern of the “southern flood and northern drought” is strengthening. Additionally, we consider that climate change potentially narrowed the temperature gap and widened the precipitation gap on a temporal and spatial scale in China. This is a preliminary study for recognizing critical meteorological elements and key regions for responding to climate change. In addition, further research should pay more attention to more meteorological elements and conduct analysis combined with the influence on the socio-economic level, the agricultural level, and the ecosystem. While in this study, the major shifts in policy or technology are not considered. This will be improved in our future study.

## Figures and Tables

**Table 1:** Extreme weather indicators.

	ID	Indicator	Definitions	Units
Extreme temperature	TN10p	Cool nights	Percentage of days when TN < 10th percentile	Day
	TX90p	Warm days	Percentage of days when TX > 90th percentile	Day
	WSDI	Warm spell duration indicator	Annual count of days with at least six consecutive days when TX > 90th percentile	Day
	CSDI	Cold spell duration indicator	Annual count of days with at least six consecutive days when TN < 10th percentile	Day
Extreme precipitation	R20	Heavy precipitation days	Annual count of days when PRCP ≥ 20 mm	Day
	CDD	Consecutive dry days	Maximum number of consecutive days with RR < 1 mm	Day
	CWD	Consecutive wet days	Maximum number of consecutive days with RR ≥ 1 mm	Day
	R95p	Very wet days	Annual total PRCP when RR > 95th percentile	mm

Note: TN is the monthly minimum value of the daily minimum temperature. TX is the monthly maximum value of the daily maximum temperature. PRCP unit = millimeters. RR is the daily precipitation amount.
